# Agonists of the Nuclear Receptor PPARγ Can Produce Biased Signaling[Fn fn4]

**DOI:** 10.1124/molpharm.124.000992

**Published:** 2024-12

**Authors:** Mariah L. Rayl, Michelle D. Nemetchek, Andrew H. Voss, Travis S. Hughes

**Affiliations:** Biochemistry and Biophysics Graduate Program (M.L.R., T.S.H.), Department of Biomedical and Pharmaceutical Sciences (M.D.N., T.S.H.), and Pharmaceutical Sciences and Drug Design Graduate Program (A.H.V., T.S.H.), University of Montana, Missoula, Montana

## Abstract

Biased signaling and ligand bias, often termed functional selectivity or selective nuclear receptor modulation, have been reported for nuclear receptor partial agonists over the past 20 years. Whether signaling differences produced by partial agonists result from less intense modulation, off-target effects, or biased signaling remains unclear. A commonly postulated mechanism for biased signaling is coactivator favoritism, where agonists induce different coactivator recruitment profiles. We find that both GW1929 (full agonist) and MRL24 (partial agonist) favor recruitment of 100 to 300 residue regions from S-motif coactivators compared with a reference full agonist (rosiglitazone), yielding 95% bias value confidence intervals of 0.05–0.17 and 0.29–0.38, respectively. Calculations based on these data indicate that GW1929 and MRL24 would induce 30% to 60% higher S-motif coactivator occupancy at the receptor compared with rosiglitazone. We compare the transcriptional effects of these same three ligands on human adipocytes using RNA sequencing and exploratory Kyoto Encyclopedia of Genes and Genomes (KEGG) pathway analysis. Only 50% (rosiglitazone) and 77% (GW1929) of all gene expression changes are shared between these full agonists after 3 hours of exposure. After 24 hours of exposure, 13/98 KEGG pathways appear more intensely modulated by rosiglitazone than GW1929 (e.g., 95% confidence interval of bias in the regulation of lipolysis in adipocytes pathway is 0.03–0.09), despite similar signaling for the remaining 85 affected pathways. Similarly, rosiglitazone has an unusually large effect on several lipid metabolism-related pathways compared with the partial agonist MRL24. These data indicate that nuclear receptor full and partial agonists can induce biased signaling, likely through differences in coactivator recruitment.

**SIGNIFICANCE STATEMENT:**

Many nuclear receptor partial agonists cause fewer adverse effects and similar efficacy compared with full agonists, potentially by inducing biased agonism. Our data support the idea that partial agonists, and a full agonist, of the nuclear receptor Peroxisome proliferator-activated receptor gamma (PPARγ) are biased agonists, causing different signaling by inducing PPARγ to favor different coactivators. These data indicate that biased agonism can occur in nuclear receptors and should be considered in efforts to develop improved nuclear receptor-targeted drugs.

## Introduction

Sixteen percent of approved drugs bind and modulate a group of transcription factors known as nuclear receptors (Santos et al., 2017). While many of these drugs provide unique standard-of-care benefits, many also cause treatment-limiting undesired effects (Burris et al., 2023). For example, multiple peroxisome proliferator-activated receptor γ (PPARγ) agonists of the thiazolidinediones (TZD) class have been approved for treating type II diabetes mellitus in humans; however, these TZDs cause treatment-limiting undesired effects (Davies et al., 2018) including edema, adipose tissue expansion, and increased bone fractures in women (Loke et al., 2009; Soccio et al., 2014). Other nuclear receptor-targeted drugs, such as immune-modulating agents that bind the glucocorticoid receptor, also cause serious adverse effects that limit their use (Sundahl et al., 2015).

A goal of current nuclear receptor drug development is to create new ligands that maintain therapeutic effects but have reduced adverse effects. Such improvements could be achieved by biased nuclear receptor agonists (Kolb et al., 2022; Burris et al., 2023) where two agonists cause different types of signaling, not just different signal intensity, through the same receptor. For example, many PPARγ partial agonists have been reported to induce equivalent insulin sensitivity but fewer undesired effects relative to TZD full agonists in animal models (for a summary of these reports, see Supplemental Table 1). Here we refer to partial and full agonism as defined by their effect on a cell-based assay using a PPAR response element reporter plasmid and overexpressed PPARγ. Full agonists produce apparently maximal luciferase production while a partial agonist produces submaximal luciferase production. Progress is being made to understand mechanisms that could underlie the improved therapeutic profile of partial agonists, including mechanisms of nuclear receptor biased signaling.

Ligand bias that results in different coactivator recruitment profiles is thought to underlie biased signaling via nuclear receptors (Burris et al., 2023). In support of this idea, we previously found that several partial agonists, including the partial agonist and non-TZD full agonist we use here [MRL24 (Acton et al., 2005) and GW1929 (Henke et al., 1998)], cause PPARγ to favor binding of one class of coactivators (S-motif coactivators) relative to a reference full agonist [the TZD rosiglitazone (Lehmann et al., 1995)]. We reported that coactivators containing an S-motif (i.e., S/TXLXXLL where X is any amino acid and S,T, and L are serine, threonine, and leucine, respectively) bind to PPARγ differently than another class of coactivators (termed N-anchored), allowing ligand bias (Kolb et al., 2022) to occur in nuclear receptors (Nemetchek et al., 2022). Unless noted otherwise, in this report we use the term ligand bias and biased agonism to describe such coactivator favoritism, and we quantify ligand bias and biased agonism using rosiglitazone and nuclear receptor binding regions of CREB-binding protein (CBP) as references.

Here we build on these peptide-based findings by showing that the full agonist GW1929 and partial agonist MRL24 induce biased recruitment, relative to rosiglitazone, of 100 to 300 residue regions of coactivators containing all their nuclear receptor binding motifs [known as receptor interaction domains/receptor interaction domain (RIDs)].

Based on these results, we hypothesized that these ligands would produce biased signaling in cells because a handful of coregulators are known to bind PPARγ; these coregulators have distinct functions (Mouchiroud et al., 2014), and each coregulator contains primarily one or the other class of LXXLL sequence. Previous work investigating biased PPARγ signaling compared a full agonist (rosiglitazone) with partial agonists (Berger et al., 2003; Motani et al., 2009; Choi et al., 2010; Tan et al., 2012); however, interpretation of these data is complicated by the fact that partial agonists could appear to modulate different genes than a full agonist because of biased agonism or because of the less intense expression modulation induced by partial agonists. We address this difficulty here by comparing signaling produced by two full agonists, GW1929 and rosiglitazone. To make this comparison, we perform RNA sequencing (RNA-seq) of human adipocyte mRNA at 3- and 24-hour postligand exposure. Our data support the idea that agonist-induced LXXLL motif preference can produce biased signaling through nuclear receptors, resulting in differential Kyoto Encyclopedia of Genes and Genomes (KEGG) pathway activation.

## Materials and Methods

### Adipocyte Cell Culture

Adipose-derived adult stem cells (ZenBio cat. #ASC-F-SL) were plated and expanded in accordance with the manufacturer’s protocols. ZenBio reports that its products are free of mycoplasma contamination. The cells were thawed and plated in the Preadipocyte Medium (Zenbio catalog #PM-1, containing Dulbecco’s modified Eagle’s medium (DMEM)/Ham’s F-12 (1:1, v/v), HEPES pH 7.4, FBS, penicillin, streptomycin, and amphotericin B) in T75 flasks. All medium was preheated prior to applying it to cells.

To differentiate adult stem cells into adipocytes, cells were first maintained in PM-1. The medium was changed after 2 days post-thaw and were split 1:2 after 4 days. Preadipocytes were plated into 6 well plates after 7 days. Twenty-four hours after plating into the 6-well plate, the medium was changed from PM-1 to the adipocyte differentiation medium without PPARγ agonist (#DM-2-PPG, containing DMEM/Ham’s F-12 (1:1, v/v), HEPES pH 7.4, FBS, biotin, pantothenate, human insulin, dexamethasone, isobutylmethylxanthine, penicillin, streptomycin, and amphotericin B) supplemented with 3 *μ*M pioglitazone. The cells remained in this medium for 7 days when they were changed to the adipocyte maintenance medium (#AM-1, containing DMEM/Ham’s F-12 (1:1, v/v), HEPES pH 7.4, FBS, biotin, pantothenate, human insulin, dexamethasone, penicillin, streptomycin, and amphotericin B). The cells were rinsed four times with AM-1 and then remained in the AM-1 medium for 1 week. See adipocyte culture photos.pptx at https://osf.io/3g2ks/ for photos of these cultures.

Mature adipocytes were then treated with drug for 3 hours, 24 hours, or 3 hours with T0070907/DMSO followed by drug for 3 hours and lysed using TRizol. RNA was isolated using the TRIzolPlus RNA Purification Kit (Invitrogen #12183555) treated with the on-column Purelink DNase Treatment (Invitrogen). Purified RNA was snap-frozen and stored at –80°C.

### PPARγ-Response-Element Transactivation Assay

HEK293T cells (RRID:CVCL_0063 purchased from American Type Culture Collection CRL3216) were stored in liquid nitrogen vapors. American Type Culture Collection reported no mycoplasma contamination was detected upon purchase; we did not perform any additional mycoplasma testing during culturing. They were thawed and placed into 9 ml of fresh complete growth medium (DMEM supplemented with 10% heat-inactivated FBS, 1% penicillin/streptomycin, and 1% 200 mM L-glutamine). Cells were centrifuged at 100 × g for 7 minutes. They were resuspended in 1 ml of complete growth medium and plated into T75 flasks with 19 ml of equilibrated medium (15 minutes in T75 flasks at 37 ˚C with 5% CO_2_). Cells were passaged at least twice post-thaw. Cells were passaged by removing the medium, rinsing twice with 7 ml of Dulbecco's phosphate-buffered saline, adding 2 ml 0.25% trypsin-EDTA, and incubating at room temperature until the cells detached from the T75 flask. They were batch transfected in T75 flasks using 4.3 *μ*g of the PPARγ plasmid, 4.3 *μ*g of the peroxisome proliferator response element (PPRE) plasmid, and 25.8 *μ*l of XtremeGene (Millipore Sigma) at a density of 3.82 * 10^6^ cells per well. Cells were removed from the plate and plated into white 384-well plates at 10,000 cells/well in 20 *μ*l per well. They incubated for 4 hours at 37°C with 5% CO_2_. Ligand was added in 20 *μ*l complete growth medium so that the cells received the final concentrations indicated in Supplemental Table 6. Cells were incubated for 24 hours and lysed using 20 *μ*l BriteLite Plus (Perkin Elmer).

The mammalian expression PPARγ plasmid and the pGL2 PPRE luciferase reporter plasmids were gifts from the Douglas Kojetin laboratory. The pGL2 PPRE luciferase reporter plasmid contains three direct repeat 1 (DR1) binding sites 5′ to the luciferase gene with the canonical binding sequence “AGGACAaAGGTCA.”

### TR-FRET Blocking Experiment with Inverse Agonist T0070907

To detect off-target binding effects, we determined the ligand concentrations required to fully block PPARγ in a TR-FRET experiment. To validate that the covalent inverse agonist T0070907 (CAS 313516-66-4) prevents rosiglitazone (CAS 122320-73-4) and GW1929 (CAS 196808-24-9) binding to PPARγ, we preblocked 6xHis-PPARγ ligand binding domain (LBD) with T0070907 for 3 hours followed by the addition of agonists for 2 hours; 0.9 nM anti-6xHis terbium antibody, 8 nM PPARγ LBD, and 400 nM CBP peptide were added to buffer [25 mM 4-morpholinepropanesulfonic acid (MOPS), 25 mM KCl, 1 mM EDTA, 0.01% fatty-acid free bovine serum albumin, 1% Tween, and 5 mM tris(2-carboxyethyl)phosphine (TCEP), pH 7.4) and plated into a black 384-well plate (Grenier Bio-one, catalog number 784076). T0070907 was added for a final concentration of 68 nM for 3 hours. DMSO was added to control wells. After 3 hours, GW1929 and rosiglitazone were added, and the plate was then incubated at room temperature in the dark for 2 hours and read on a plate reader using 380 nm excitation and reading emissions at 488 and 528 nm.

### Protein Purification

6xHis-tagged proteins were purified from BL21-DE3 E. coli (NEB: C2527). PPARγ full length (FL) (isoform 2, 1-505), retinoic acid receptor alpha (RXRα) FL (1-462), and PPARγ LBD (231-505) were grown in autoinduction both, terrific broth, and LB broth, respectively. 6xHistidine-tagged RIDs were grown in LB. After growing to an optical density of 0.8 at 37 deg, RXR*α* and PPARγ LBD were induced with 0.5 mM isopropyl β-D-1-thiogalactopyranoside at 22deg overnight. 6xHis coregulator RIDs CBP_1-127_, PGC1*α*_100-220_, and MED1_557-870_ were grown in LB broth and induced with 0.5 mM isopropyl β-D-1-thiogalactopyranoside at 37°C for 3 hours. Pellets were collected at 10,000xg and frozen until purification.

PPARγ FL, RXR*α* FL, and PPARγ LBD, E. coli pellets were thawed and homogenized using an immersion blender and lysed with an Avestin c5 emulsiFlex (Ottawa, Canada) in 1x lysis buffer containing 50 mM KPO4 pH 8.0, 300 mM NaCl, 1 mM TCEP, and 15 mM imidazole. Lysate was clarified at 19,000xg for 45 minutes, 0.45 micron filtered, run over two Histrap FF columns in tandem, and eluted with lysis buffer containing 500 mM imidazole. PPARγ LBD was further purified by removal of the 6xHis tag with TEV 1:40 w/w incubation overnight at 4°C followed by a second Histrap FF column to remove tag and TEV. PPARγ FL, RXR*α* FL, and cleaved PPARγ LBD were all run though a 16/600 Superdex 200 (PPARγ FL and RXR*α* FL) or Superdex 75 (PPARγ LBD) in buffer containing 25 mM MOPS pH 7.4, 300 mM KCl, and 1 mM TCEP prior to binding experiments.

For coregulator RID purification, cell pellets were homogenized using an immersion blender into pure 18M*Ω* water using a ratio of 1 g cell pellet per 10 ml water. The homogenate was then boiled in 50 mL conical tubes for 30 minutes to lyse cells and denature unwanted proteins, as done in published works for intrinsically disordered proteins (Livernois et al., 2009). Boiled lysate was cooled on ice for 5 minutes before transferring to new 50 mL conical tubes for clarification at 19,000xg. 10x lysis buffer was added to lysate prior to filtering with a 0.45 micron filter. Protein was purified using a Histrap FF column (Cytiva). Protein purity was verified using SDS-PAGE and proteins were verified to have the correct mass by quadrupole time-of-flight mass spectrometry (Supplemental Fig. 1).

### Competitive Anisotropy

Competitive anisotropy was used to measure the K_i_ of coregulator RIDs for PPARγ FL. All proteins were buffer exchanged into buffer containing 25 mM MOPS pH 7.4, 25 mM KCl prior to anisotropy experiments.

Binding experiments were performed in black 384-well plates (Grenier Bio-One, catalog number 784076) by adding 8 microliters of PPARγ FL and ligand to 1600nM (800nM final concentration) and 5FAM-CBP peptide (Invitrogen PV4596) to 100nM (50nM final concentration) in assay buffer containing 25 mM MOPS pH 7.4, 25 mM KCl, 10 mM TCEP, 0.02% Tween 20, 0.02% fatty-acid free bovine serum albumin fraction V (EMD Millipore, catalog number 126575) to all wells. Heterodimer conditions additionally contained equimolar amounts of PPARγ FL, RXR*α* FL, and dsDNA containing the SULT2A1 gene PPRE (5′-GTAAAATAGGTGAAAGGTAA - 3′) synthesized by IDT DNA. Eight microliters containing a CBP 1-127, peroxisome proliferator-activated receptor gamma coactivator-related protein 1 (PGC1α) 100–220, or mediator of RNA polymerase II transcription subunit 1 (MED1) 557–870 ranging from concentrations of approximately 1uM – 370 μM in buffer containing 25 mM MOPS pH 7.4, 25 mM KCl was added to each corresponding well in a 384-well plate. Each binding experiment contained 24 concentration points, 2 technical replicates per dilution replicate, and 8 dilution replicates. Once all wells contained the full 16 microliters of sample, they were spun at 500xg for 1 minute to reduce bubbles from pipetting. Plates were incubated at room temperature in the dark for 2 hours as 2 hours was necessary to reach equilibrium (Supplemental Fig. 8). Plates were then read using an FP compatible cube in a Synergy H1 platereader (Biotek), and anisotropy values of the FL-CBP peptide were calculated from parallel and perpendicular intensities using the anisotropy equation = (I_II_ − I_⊥_)/(I_II_ + 2I_⊥_). EC_50_ values were calculated in Prism using the [Agonist] versus response – Variable slope (four parameters) equation with ROUT coefficient Q = 1% for outlier removal.

K_i_ values for each coregulator RID were calculated from EC_50_ values as reported previously (Nemetchek et al., 2022) using a modified Huang et al. equation (Auld et al., 2012):
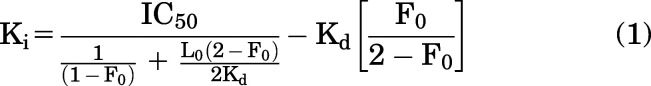
where F_0_ is the fraction of tracer (5FAM-labeled peptide) bound and L_0_ is total tracer concentration. Concentrations of fraction bound tracer (FL-CBP peptide) were calculated using a custom python 2.7 script available on the Center for Open Science repository at osf.io/m98we. Fraction-bound values can also be calculated using a web-based script: https://www.wolframalpha.com/widgets/view.jsp?id=3f9ea5a91e04b49316f83f8143fffa30.

Bias values of PPARγ bound to each ligand for a coregulator RID were calculated as described previously (Nemetchek et al., 2022). Sigma values were calculated for each test ligand (GW1929, MRL-24 CAS 93794-17-7) with respect to reference ligand rosiglitazone:
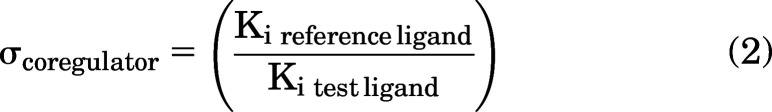


Ligand bias for each coregulator was calculated with respect to CBP_1-127_ as a reference:
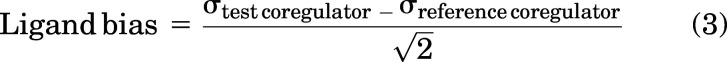


The underlying data and calculations for ligand bias can be found in the supplemental dataset Datafile1.xlsx.

### RNA-Seq

Library preparation and mRNA sequencing was done by Novogene Corporation using Illumina PE150 platforms. Novogene also removed the adaptors and low-quality reads from the sequencing data. The number of replicates sequenced for each ligand can be found in Supplemental Table 3.

### Differential Expression Analysis

Novogene returned cleaned raw data. Reads were aligned to the GRCh38 genome using HISAT2 v. 2.2.0 (Kim et al., 2019). FastQC (http://www.bioinformatics.babraham.ac.uk/projects/fastqc/) was run on the resulting BAM files. SAM files were converted to BAM files using SAMtools v. 1.15.1 (Danecek et al., 2021). Transcript assembly was performed using StringTie v. 2.2.1.Linux_x86_64 (Pertea et al., 2015, 2016) with GCA_000001405.15_GRCh38_full_analysis_set.refseq_annotation.gtf as the reference annotation. Gffread v. 0.12.7.Linux_x86_64 (Pertea and Pertea, 2020) was used to make the annotation readable by StringTie. Only mRNA annotations were included. The resulting files were converted to a table with prepDE.py available from http://ccb.jhu.edu/software/stringtie/index.shtml?t=manual. Differential expression was determined with DESeq2 v. 1.32.0 using the following R packages in RStudio: DESeq2 v. 1.32.0 (Love et al., 2014), ClusterProfiler v. 4.0.5 (Wu et al., 2021), DOSE v. 3.19.3.992 (Yu et al., 2015), enrichplot v. 1.13.1.992 (R package version 1123), ggplot2 v. 3.3.6 (Wickha, 2016), org.Hs.eg.db v. 3.13.0 (R package version 3.8.2), and AnnotationDbi v. 1.54.1. An adjusted *P*value cutoff of 0.05 was imposed to identify genes that were statistically significantly differentially expressed. RNA-seq data were deposited under GSE266107.

### KEGG Pathway Bias Calculation

As part of the bias value calculation, a sigma value was calculated. When calculating signaling bias, if the test (GW1929) and reference ligand (rosiglitazone) affect the AMPK pathway equally, then sigma will equal zero. The sigma values for the AMPK KEGG pathway are 0.076 and –0.084 at 3 and 24 hours, respectively, for GW1929, indicating that both ligands affect the expression of the genes included in the KEGG AMPK pathway nearly the same, as expected for full agonists. All differentially expressed genes were used to create a dataset of affected KEGG pathways utilizing clusterProfiler in R. Differentially expressed genes were grouped by treatment time and ligand for determination of enriched pathways using compareCluster within clusterProfiler with an adjusted *P* value cutoff of 0.05. The AMPK pathway was selected as the reference pathway with rosiglitazone as the reference ligand. Bias for each gene within a pathway was calculated as follows:
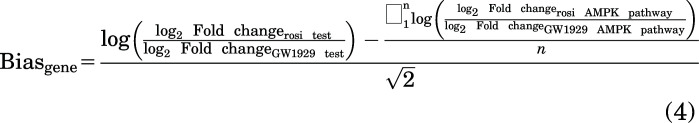


[Adapted from Rajagopal et al. (2011) for use with RNA-seq data].

Pathways where the 95% confidence interval of the bias values for the genes in that pathway did not include zero was considered a biased pathway.

Other equations are as follows:
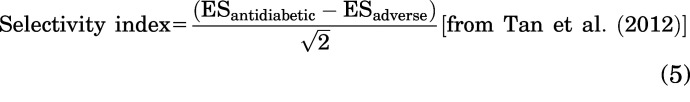

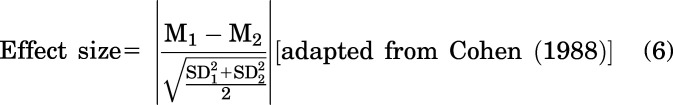


### DR1 Enrichment Analysis

We analyzed the genomic sequence 10 kilobases upstream of the transcription start site (TSS) for all the transcripts included in the differential expression analysis. Sequences were obtained from the UCSC Genome Browser (Nassar et al., 2023) using the Table Browser tool (Karolchik et al., 2004). The PPARγ:RXR*α* binding motif (GGGTCAAAGGTCA) was obtained from JASPAR (Rauluseviciute et al., 2024). The sequences were queried for the motif using HOMER (Heinz et al., 2010) using a motif score cutoff of 7.350619126 calculated by the sum of the log-odds probability for each nucleotide in the sequence. Differentially expressed transcripts were analyzed for frequency of containing a PPARγ:RXR*α* binding motif within 10 KB of the TSS.

### Other

Venn diagrams were created using http://bioinformatics.psb.ugent.be/webtools/Venn/.

### Occupancy Calculation for Fig. 1D

See Supplemental Methods 1 for a derivation of the formula we used to calculate occupancy under conditions of two coregulators competing for binding to the same binding site on PPARγ. We estimated concentrations of the transcription cofactors (PGC1, CBP, and MED1) and PPARγ using numbers found in the Bionumbers database (https://bionumbers.hms.harvard.edu/search.aspx) and Biggin (2011). In these calculations, we assume that PPARγ is at a much lower concentration than the coregualtors. We estimate the concentration of PPARγ to be 50 nM and the coregulators between 150 and 1500 nM based on information in these sources (see Datafile1.xlsx sheet panel d for estimates based on the cited sources).

## Results

### Ligand Bias Observed with Coregulator RIDs

**Fig. 1.**
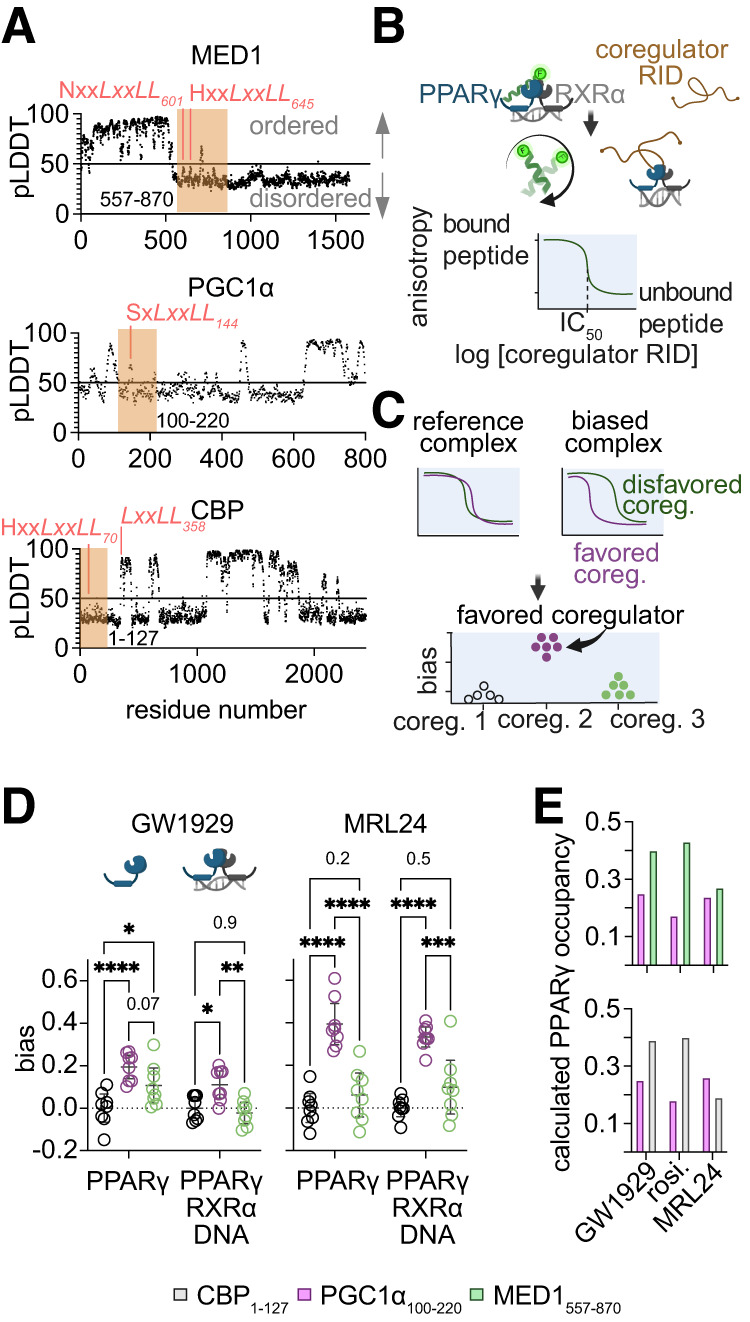
A full agonist and partial agonist cause PPARγ to favor binding PGC1*α*_100-220_. (a) The Alphafold pLDDT, a prediction of ordered structure, per amino acid of the coregulators used here. pLDDT < 50 indicates a region that is likely intrinsically disordered (Akdel et al., 2022). Nuclear receptor binding motifs are noted in peach. The subscript represents the residue number of the first leucine in the LXXLL motif. (b) Competitive anisotropy method. Purified coregulator RIDs compete with a fluorescently labeled CBP_70_ peptide for binding to PPARγ, producing an IC_50_ value (and K_i_). (c) Bias values are calculated from K_i_ values. (d) Bias values, 95% confidence interval, and mean are displayed for PPARγ-ligand complexes or these complexes bound to RXR*α* and a dsDNA PPRE sequence from the SULT2A1 gene (*n* = 8). Rosiglitazone and CBP_1-127_ are used as references. Significance of differences were analyzed using a two-way ANOVA followed by Tukey’s multiple comparison’s test. *P* values for each comparison are shown. (e) Calculated occupancy of PPARγ in cells using affinity data underlying panel (d) and known cellular transcription factor and cofactor concentrations. Data points, calculations, assumptions, and raw data can be found in Datafile1.xlsx and Supplemental Methods 1. BioRender.com was used to generate part of this figure.

We previously reported that GW1929 is a biased agonist relative to rosiglitazone as it causes PPARγ to favor binding S-motif containing coregulator peptides relative to a CBP peptide (Nemetchek et al., 2022); however, coregulators are large proteins that typically contain large intrinsically disordered regions (Fig. 1a) containing multiple nuclear receptor binding motifs. We hypothesized that GW1929 and partial PPARγ agonists would also induce biased recruitment of larger regions of these coregulators. To test this, we recombinantly expressed and purified RIDs of the coregulators MED1 (two LXXLL motifs): PPARγ coactivator 1-alpha (PGC1*α*; one LXXLL motif) and CBP (one LXXLL motif) from E. coli (Supplemental Fig. 1 and Supplemental Table 2). These RIDs contain all LXXLL motifs present in MED1 and PGC1*α* and the only LXXLL motif in CBP, which binds PPARγ (Broekema et al., 2019).

To measure the affinity of these RIDs for PPARγ, we developed a competitive binding assay using fluorescence anisotropy (Fig. 1b). We measured the competitive dissociation constant (K_i_) of each RID for ligand-bound PPARγ and for ligand-bound PPARγ complexed with retinoic acid receptor alpha (RXR*α*) and dsDNA containing the SULT2A1 peroxisome proliferator response element (PPRE) (Runge-Morris et al., 2013). Our past work using LXXLL peptides has demonstrated that RXR*α*’s contribution to the binding of these coregulator LXXLL motifs is minimal, as the dissociation constant of a heterodimer containing a mutant PPARγ that binds LXXLL motifs poorly (PPARγE471L/RXR*α* bound to either MRL24 or GW1929) is 10 to 100x weaker than the affinity of the wt PPARγ/RXR*α* heterodimer (bound to either MRL24 or GW1929) for the LXXLL motifs contained in the CBP, MED1, and PGC1*α* RIDs (Nemetchek et al., 2022). Bias values for each ligand were calculated using an established equation for ligand bias (Kolb et al., 2022) as we did previously for peptide-receptor interactions (Fig. 1c) using rosiglitazone and the CBP RID (CBP residues 1–127; CBP_1-127_) as references. We found that both GW1929 and MRL24 promote enhanced interactions between PPARγ and PGC1*α*_100-220_ relative to rosiglitazone resulting in statistically significant bias (95% confidence interval of 0.05–0.17 and 0.29–0.38, respectively; Fig. 1d and Datafile1.xlsx). These bias values are similar to what we observed previously using peptides (Nemetchek et al., 2022), with MRL24 inducing stronger bias than GW1929 and both of these ligands causing the PPARγ-RXR*α*-DNA complex to disfavor RIDs containing LXXLL motifs that bind in an N-anchored manner to PPARγ (i.e., CBP_1-127_ and MED1_557-870_). This bias is expected to reduce recruitment of CBP, MED1, and other N-anchored coregulators and increase recruitment of PGC1*α* and other S-motif containing coregulators to PPARγ at best-estimate cellular transcription factor and coregulator concentrations (Milo et al., 2009; Biggin, 2011) (Fig. 1e and Datafile1.xlsx). Given these data and considering the differing physiological roles of these coregulators (Mouchiroud et al., 2014; Narita et al., 2021), we expect GW1929 and MRL24 to produce biased signaling relative to rosiglitazone in cells.

### GW1929 and MRL24 Produce Biased Signaling in Adipocytes

To determine whether this ligand bias could result in biased signaling in cells, we treated human adipocytes with GW1929, MRL24, and the reference agonist (rosiglitazone) and isolated RNA for deep sequencing (RNA-seq). We use adipocytes because PPARγ is highly expressed in adipocytes and is the primary regulator of adipogenesis (Tontonoz and Spiegelman, 2008; Fagerberg et al., 2014). Both GW1929 and rosiglitazone were recently included in a larger chemogenomics ligand set for the NR1 receptors because they are structurally dissimilar, have no detectable off-target effects, and do not activate other NR1 receptors, except for similar weak activation of PPAR*α* and PPAR*δ* at relatively high concentration (Isigkeit et al., 2024). To further minimize the chance of off-target effects, we used relatively low ligand concentrations—at least 100-fold lower than the dissociation constant for PPAR*α* and PPAR*δ* (361 nM rosiglitazone and 28 nM GW1929); however, we expect these concentrations to provide > 90% receptor occupancy as they are 30–300 fold greater than the dissociation constant of rosiglitazone and GW1929 for PPARγ (Shang and Kojetin, 2021; Nemetchek et al., 2022). In addition, we previously showed that a higher 2.*5 μM* concentration of rosiglitazone and GW1929 did not produce consistently higher or lower luciferase production compared with the lower concentrations used here in three independent cell-based assays, indicating that the lower doses are saturating (Nemetchek et al., 2022). Because GW1929 and rosiglitazone cause apparently maximal activation of a PPRE reporter, we expect GW1929 and rosiglitazone to change the same genes to similar degrees if biased coactivator RID recruitment does not produce biased signaling. To test the prespecified null hypothesis that biased coactivator RID recruitment does not produce biased signaling, we performed two independent experiments: one at 3 hours and another at 24 hours post-treatment. The 3-hour and 24-hour experiments used different pools of adult stem cells obtained from multiple subcutaneous depots (Supplemental Table 3). Replicates within each of these experiments started from the same pool of adult stem cells; however, after initial expansion, the adult stem cells were differentiated and maintained independently for each replicate over the course of 2 weeks, after which they were treated with ligand or vehicle. The 3-hour timepoint highlights direct PPARγ effects, is early enough to correlate with nascent RNA production 10 to 30 minutes post-treatment (Step et al., 2014), and was used previously in similar work (Haakonsson et al., 2013). We expected the 24-hour timepoint to detect more ligand effects, including effects secondary to activation of PPARγ.

**Fig. 2.**
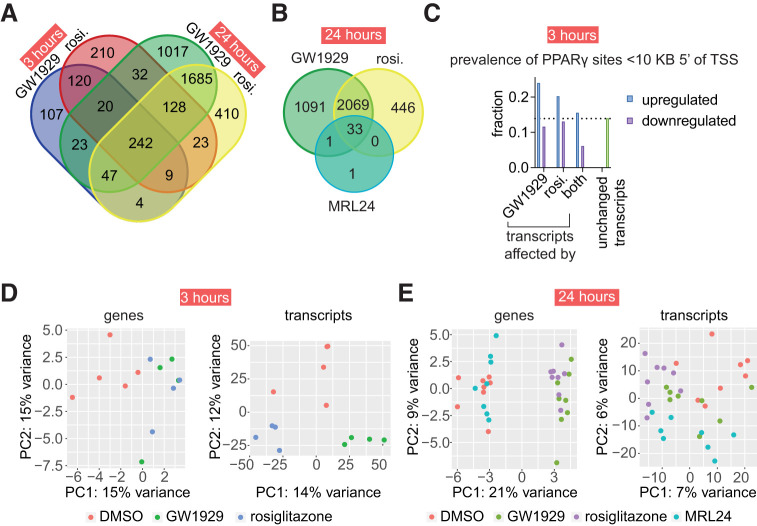
GW1929 affects the expression of different genes than rosiglitazone through modulation of PPARγ. (a-b) At both 3 and 24 hours, each full agonist differentially expresses unique sets of genes (adjusted *P* value < 0.05). At 24 hours, MRL24 affects the expression of relatively few genes. (c) Prevalence of PPARγ DNA binding sites within 10 kilobases upstream (5′) of transcript start sites of transcripts that were differentially expressed by only GW1929, only rosiglitazone, both agonists, or neither (unchanged transcripts). (d-e) Principal component analysis of all differentially expressed genes and transcripts at 3 and 24 hours post ligand exposure in human adipocytes. This analysis used the r log method. See Supplemental Fig. 3 for results using variance stabilizing transformation. See Datafile2_3h.xlsx and Datafile2_24h.xlsx for gene names and underlying data. The experiments analyzed in this figure used 4 replicates (3-hour data) or 8 replicates (24-hour data).

Preplanned differential expression analysis revealed that, at both timepoints, these full agonists change the expression of many of the same and different genes (Fig. 2a, Datafile2_24h.xlsx, and Datafile2_3h.xlsx). The results from the 3-hour adipocyte experiment were used to conduct a power analysis using the ssizeRNA v 1.3.2 package in R (Bi and Liu, 2016), which we used to determine the number of replicates needed to limit false negatives in the 24-hour adipocyte experiment (Supplemental Fig. 9). The 3-hour experiment used four replicates rather than the eight replicates used in the 24-hour experiment, which could contribute to the increase in differentially expressed genes detected at 24 hours (Fig. 2).

Analysis of differentially expressed genes revealed that 181 genes were affected by only GW1929 at the 3-hour timepoint. At 24 hours, gene expression for 47 of these 181 genes became affected by both full agonists, 23 continued to be affected only by GW1929, 4 were affected only by rosiglitazone, and 107 were unaffected by either agonist. Similarly, the expression of 393 genes was uniquely changed by rosiglitazone at the 3-hour timepoint. At 24 hours, gene expression for 128 of these 393 genes became affected by both full agonists, 23 continued to be only affected by rosiglitazone, 32 were only affected by GW1929, and 210 were unaffected by either agonist (Fig. 2a). Principle component analysis of all differentially expressed transcripts (and expression of associated genes) indicates that at 3 hours and 24 hours GW1929 and rosiglitazone have different transcriptional effects (Fig. 2).

The genes whose expression was affected by just one of the agonists at 3 hours commonly became mutually affected or unaffected by either agonist at the 24-hour timepoint. Possible biological causes for gene expression being modulated by one agonist at 3 hours and both at 24 hours include one agonist impacting gene expression more or more rapidly than the other agonist because of differences in CBP recruitment (Ma et al., 2021) or pharmacokinetic differences between agonists (which we address later). About 50% of the genes whose expression is modulated by rosiglitazone at 3 hours are not affected at 24 hours. This shift from modulated to not affected is consistent with our analysis of deposited RNA-seq data derived from treatment of mouse 3T3L1 adipocytes with rosiglitazone [Gene Expression Omnibus dataset GSE56747 (Step et al., 2014); Supplemental Fig. 2].

Twenty-three genes whose expression was affected by just one agonist at the 3-hour timepoint continued to be uniquely affected by the same agonist at the 24-hour timepoint. The 3-hour and 24-hour experiments were independent experiments carried out months apart using different pools of donor samples (Supplemental Table 3). These persistent, agonist-specific effects support the idea that PPARγ agonists, and nuclear receptor agonists in general, can produce biased signaling.

We also tested the effect of the partial agonist MRL24 on adipocyte transcription at 24 hours in an exploratory experiment. Calculated *P* values cannot be interpreted as hypothesis testing but only as descriptive for such exploratory experiments. We previously found that MRL24, like GW1929, favors S-motif containing coregulator peptides (Nemetchek et al., 2022). In addition, MRL24 causes less heart and body weight gain in mice and rats than rosiglitazone while providing similar insulin sensitization effects (Acton et al., 2005). At 24 hours post-treatment, MRL24 causes differential expression of very few genes compared with the agonists, and nearly all these changes are shared with the two full agonists (Fig. 2b and Datafile2_24h.xlsx). Overall, the partial agonist MRL24 regulates a subset of all genes regulated by the full agonists.

### Biased Signaling Originates From Ligand Bias

We identified four potential explanations for the putative biased signaling we observed between GW1929 and rosiglitazone: 1) uniquely affected genes and signaling differences are caused by off-target effects (i.e., activation of non-PPARγ receptors), 2) one agonist modulates the expression of all or most genes more strongly than the other making transcriptomic changes better detected for that agonist, 3) one agonist requires less time to engage PPARγ in the nucleus, or 4) the ligands produce different signaling via PPARγ (ligand bias). We performed further analysis and additional experiments to test hypotheses related to explanations 1 through 3, which do not invoke ligand bias.

To help determine whether biased signaling occurs through activation of PPARγ and not off-target receptors, we analyzed the sequence upstream of transcript start sites and found that PPARγ binding sites (i.e., DR1 DNA sequences) are overrepresented in transcripts upregulated by only GW1929 and only rosiglitazone compared with unchanged transcripts at 3 hours (Fig. 2c). These findings indicate that on-target PPARγ effects underlie the observed differences in PPARγ signaling for upregulated transcripts. To further determine the prevalence of off-target binding effects, we used the covalent inverse agonist T0070907 to block ligand binding to PPARγ (Meylan et al., 2021) before addition of GW1929 and rosiglitazone. We first confirmed that T0070907 blocks modulation of PPARγ by GW1929 and rosiglitazone (Supplemental Fig. 5). We added T0070907 to adipocytes, added GW1929 or rosiglitazone 3 hours later, and then harvested mRNA 3 hours after addition of the agonists and performed sequencing (Supplemental Fig. 4). Seventy-six percent (729) of the genes that were differentially expressed at 3 hours (Fig. 2) were also statistically significantly chemically blocked by T0070907 pretreatment (Supplemental Figs. 4 and 6 and Datafile2_3h.xlsx), indicating that the expression of these genes is changed by ligand binding to PPARγ and not another receptor, corroborating the clean targeting profile for these two ligands in published cell-based assays (Isigkeit et al., 2024).

To test whether one full agonist modulates the expression of all or most genes more strongly than the other, we compared the effect sizes of all transcriptional changes induced by GW1929 and rosiglitazone. At both 3 and 24 hours, neither ligand produced a statistically significant difference in effect size ([mean effect size; 95% confidence interval] 3 hours. GW1929 [2.45; 2.36-2.52], 3 hours. rosi. [2.24; 2.19-2.30], 24 hours. GW1929 [1.98; 1.95-2.01], 24 hours. rosi. [1.98;1.95-2.01]), indicating likely saturation of PPARγ at the concentrations used in the RNAseq experiment (Supplemental Fig. 4). This lack of difference in effect size is consistent with our previously published data showing no difference between these low concentrations and 2.*5 μM* on luciferase production in a PPRE reporter assay (Nemetchek et al., 2022).

Finally, to determine whether one agonist engages PPARγ and produces transcriptional changes faster than the other, we used a PPRE cell-based assay to evaluate when these agonists first cause statistically significant transcriptional changes. In two independent experiments, we found that neither agonist consistently induced greater luciferase production than the other at 3, 4, 5, 6, or 24 hours post-ligand treatment (Supplemental Fig. 4 and 7 and Datafile4.xlsx).

Together these data indicate that ligand bias underlies the different transcriptional effects observed between GW1929 and rosiglitazone.

### Rosiglitazone Modulates Specific KEGG Pathways More Than GW1929

**Fig. 3.**
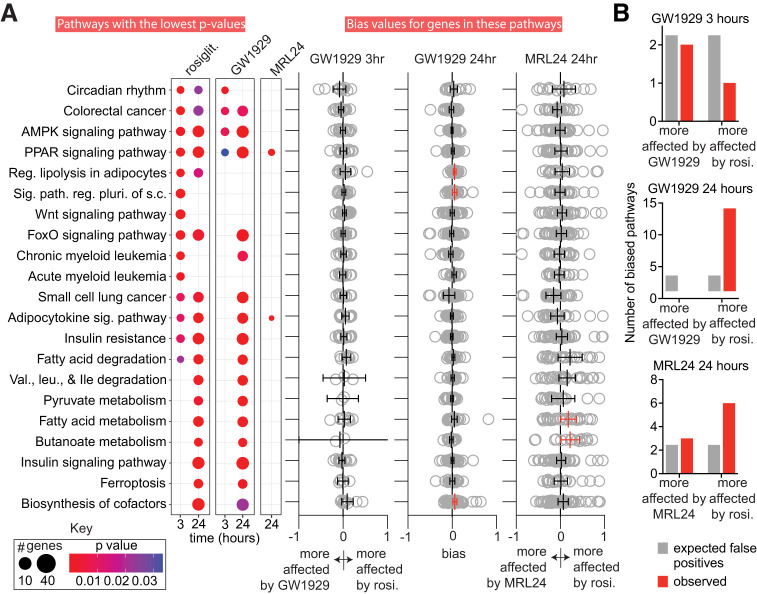
GW1929 and MRL24 produce biased signaling along specific KEGG pathways. (a) Top 21 pathways that are affected by at least one ligand according to analysis by ClusterProfiler are shown. The number of affected genes in each pathway and the adjusted *P* value for pathway modulation is indicated by the size and color of the dot (see Key). The right side of panel (a) shows bias values for all genes in each affected pathway. The mean and 95% confidence interval (CI) for the bias values are also indicated. Biased pathways where the 95% CI does not overlap zero are highlighted in red. To reduce whitespace, 1 and 19 large bias values are not shown for the GW1929 24 hours and MRL24 24 hours, respectively. (b) Number of putative biased pathways observed vs. number of biased pathways expected due to chance is displayed. See Datafile3.xlsx for underlying data. The experiments analyzed in this figure used four replicates (3-hour data) or eight replicates (24-hour data).

To determine whether the agonists modulate signaling pathways to different degrees, we performed exploratory analysis of these RNA-seq results. We identified treatment-affected KEGG pathways at each timepoint using enrichKEGG and compared affected pathways using the compareCluster function in clusterProfiler (Yu et al., 2012). The 21 KEGG pathways and categories that are the most likely affected by any ligand (i.e., those with the lowest adjusted *P* values) are shown in Fig. 3a. Many of these pathways are expected to be affected by PPARγ-binding ligands as PPARγ controls many aspects of lipid metabolism. The full agonists affect many of the same pathways at the 24-hour timepoint (36 pathways have an adjusted *P*value of < 0.05 for both full agonists). Thirty-seven pathways are affected by at least one agonist at 24 hours (adjusted *P*value < 0.05; Fig. 3a and Datafile2_24hr.xlsx). MRL24 affects only two pathways: the PPARγ and adipocytokine signaling pathways. Mirroring the putative timing differences in changes of the expression of individual genes, rosiglitazone affects many more pathways than GW1929 at 3 hours (41 vs. 4; Fig. 3a and Datafile2_3hr.xlsx). While this difference could result from differences in ligand pharmacokinetics or differences in signaling, as stated previously we see no evidence of different ligand pharmacokinetics (Supplemental Figs. 4 and 7).

To better understand whether GW1929 and MRL24 cause biased signaling along specific pathways compared with rosiglitazone, we calculated bias values (see Methods) for all affected KEGG signaling pathways and categories. Such bias values let us determine if GW1929 or MRL24 induce different signaling intensity along individual pathways compared with rosiglitazone and a reference pathway. We used the AMPK pathway as the reference pathway as it is the most consistently affected by both GW1929 and rosiglitazone in the ClusterProfiler output and is a well-known PPARγ target pathway, and because both agonists affect this pathway very similarly (i.e., *σ* values are small; *σ* = 0.076 and –0.084 at 3 and 24 hours, respectively). Ninety-eight pathways were identified as affected by at least one of the drugs at either timepoint. We calculated the relative impact of the three ligands on these 98 pathways, generating a bias value for all genes in each pathway for each agonist. Genes whose expression is affected more strongly by rosiglitazone (either more upregulated or more downregulated) yield a positive bias value, while genes whose expression is more affected by the comparator ligand (GW1929 or MRL24) yield a negative bias value.

To gain a sense of the overall impact of the ligands on each pathway, we grouped these gene bias values by pathway and timepoint and then calculated the 95% confidence interval of the average bias for each pathway. We considered pathways whose 95% confidence interval did not cross zero as biased (Fig. 3a and Datafile3.xlsx). By chance we would expect two to three pathways to appear more strongly regulated by rosiglitazone, GW1929, or MRL24 for each comparison. Only the 24-hour timepoint yielded more biased pathways than expected from chance (Fig. 3b). Comparison of GW1929 and rosiglitazone signaling at 24 hours indicates that 13 KEGG pathways are more strongly modulated by rosiglitazone, while none are more affected by GW1929. A comparison of MRL24 and rosiglitazone indicates that six pathways are more impacted by rosiglitazone while three are more affected by MRL24 (Fig. 3b and Datafile3.xlsx). Consistent with GW1929 and MRL24’s efficacy in blood glucose correction (Henke et al., 1998; Acton et al., 2005), the insulin sensitization and signaling and adipocytokine pathways are modulated to a similar degree compared with rosiglitazone. Interestingly, the regulation of lipolysis in adipocytes and the fatty acid metabolism pathways are modulated more strongly by rosiglitazone than GW1929 and MRL24 respectively (Fig. 3a, Datafile3.xlsx).

Tan et al. previously identified groups of genes associated with antidiabetic (blood glucose correction) and adverse effects (cardiac hypertrophy) in rodents and described a selectivity index that quantitates whether a PPARγ-binding ligand has relatively greater antidiabetic or adverse effects (Tan et al., 2012). A higher selectivity index indicates more antidiabetic and less adverse effects. We were able to identify human orthologs for most of the genes in these two sets (Supplemental Table 4). Using these slightly reduced gene sets, we found that GW1929 had a higher selectivity index than rosiglitazone at 3 hours. At 24 hours both GW1929 and rosiglitazone switched to similar negative selectivity indexes, while MRL24 produced a positive index at 24 hours (Supplemental Table 5). This supports the findings by Tan et al. that partial agonists have a higher selectivity index compared with full agonists at 24 hours post-treatment (Tan et al., 2012). It also suggests that GW1929 produces different signaling in adipocytes than rosiglitazone.

Together these data indicate that the full PPARγ agonists rosiglitazone and GW1929 induce different signaling through the same nuclear receptor, making GW1929 a biased agonist relative to rosiglitazone. Our data, and known structural differences between partial agonist and rosiglitazone-bound PPARγ, indicate that partial PPARγ agonists change a subset of the signaling pathways affected by full agonists and act as biased agonists relative to rosiglitazone.

## Discussion

Nuclear receptors modulate the expression of a wide array of genes in different tissues. The demonstrated ability to fine-tune signaling through nuclear receptors via biased agonists would change new nuclear receptor drug development. This idea, often referred to as selective nuclear receptor modulation or functional selectivity, has been explored, primarily through the development of novel partial nuclear receptor agonists, for over 20 years (Burris et al., 2013, 2023). Research focused on quantifying biased signaling and determining the cause and consequences of such signaling is needed to establish whether biased nuclear receptor agonists are likely to provide improved therapeutic effects relative to currently prescribed drugs.

This report provides a step toward this goal by demonstrating that nuclear receptor agonists can induce different signaling through the same receptor and not just different intensities of signaling [i.e., they can cause biased signaling (Kolb et al., 2022)]. The reference full agonist (rosiglitazone) produced apparently stronger signaling in about a tenth of all agonist-affected KEGG pathways at 24 hours postexposure (Fig. 3) despite no difference in overall intensity of gene expression modulation relative to the compartor full agonist (GW1929, Supplemental Fig. 4). We found that a partial agonist (MRL24) had an unusually weak effect on approximately six KEGG pathways. While the apparent weak signaling in two or three of these six pathways is likely due to noise, three or four are not. Interestingly, three of the weakly modulated pathways are related to fatty acid metabolism (fatty acid elongation, biosynthesis of unsaturated fatty acids, and fatty acid metabolism; Data3.xlsx), which may explain why, unlike rosiglitazone, MRL24 does not induce weight gain (Acton et al., 2005).

A mechanism that could underlie such biased signaling is biased coactivator recruitment. It is well-known that agonists increase binding of PPARγ to LXXLL motif containing transcriptional coregulators (Weikum et al., 2018) (where L is Leucine and X is any amino acid); however, we previously found that residues N-terminal to the LXXLL motif are also important to coregulator-PPARγ interaction. Coregulator peptides with the HXXLXXLL motif (where H is Histidine) interact with PPARγ Helix 4 (a binding mode termed N-anchored). A similar interaction is found in other nuclear receptors between residues 2 or 3 residues N-terminal to the first leucine of the LXXLL helix and Helix 4. In contrast, crystal structures and mutational analysis show that coregulator peptides with S/TXLXXLL or SXXLXXLL motifs, or no discernible motif lack this interaction (Nemetchek et al., 2022). We have also found that partial agonists induce different PPARγ structures compared with TZDs such as rosiglitazone (Chrisman et al., 2018; Heidari et al., 2019). These different agonist-dependent receptor structural ensembles cause the receptor to favor peptides from one LXXLL class over the other (Nemetchek et al., 2022). Our previously published structural model of ligand bias in PPARγ (Nemetchek et al., 2022) predicts that most partial agonists are biased agonists relative to rosiglitazone because they do not affect helix 12 structure as much as full agonists (Bruning et al., 2007)). Structural differences between GW1929-PPARγ and rosiglitazone-PPARγ that could lead to ligand bias are less obvious; however, differences in Helix 3 stabilization may partially underlie the ligand bias observed between these two full agonists (Nemetchek et al., 2022).

Here we find that the same agonists that cause biased signaling relative to rosiglitazone also induce biased coactivator RID recruitment relative to rosiglitazone (Fig. 1). Previously published GST pull-down, GAL4 reporter, mammalian or yeast two-hybrid, and time-resolved fluorescence resonance energy transfer assay data indicated, qualitatively, that non-TZD PPARγ agonists can cause biased coactivator or coactivator RID recruitment relative to rosiglitazone (Supplemental Table 1). One of these reports contains data that indicates a GW1929-like full agonist (farglitazar) favors PGC1*α* recruitment relative to rosiglitazone and CBP_1-453_ (Liu et al., 2011). Furthermore, PPARγ phosphorylation is modulated by coregulator recruitment (Li et al., 2011), indicating that nuclear receptor post-translational modification differences that result in signaling differences (Choi et al., 2011) could originate from differences in coregulator recruitment. Together, these reports, our data, and our published structural model of biased agonism support the idea that PPARγ partial and full agonists (and likely other nuclear receptor agonists) can cause biased coactivator recruitment that results in biased signaling relative to rosiglitazone.

Although ligand-induced coactivator favoritism is the most often mentioned possible mechanism of nuclear receptor biased signaling, other mechanisms could produce biased signaling. PPARγ is thought to produce most signaling while heterodimerized with RXR*α* or RXR*β* (Lam et al., 2017), raising the possibility that ligands could affect heterodimerization affinity, leading to biased signaling. Recent work has also found a subset of PPARγ target genes with greater dependence on an extended 5′ DNA binding motif (Madsen et al., 2022), making it possible that ligand-induced changes to the PPARγ DNA binding domain (de Vera et al., 2017) could result in biased signaling. An estrogen receptor *α* ligand (fluorescently labeled tamoxifen) partitions specifically into MED1-containing condensates (Klein et al., 2020), supporting the idea that some nuclear receptor ligands could have a higher partition coefficient in select transcriptional condensates or hubs (Ferrie et al., 2022), leading to differential nuclear receptor occupancy at individual genes and biased signaling.

Nuclear receptor agonists induce unique PPARγ structural ensembles (Hughes et al., 2012; Chrisman et al., 2018; Heidari et al., 2019). Such unique agonist-induced structures in areas of PPARγ outside of the coregulator binding surface could produce agonist-specific interaction with post-translational modification machinery (Ahmadian et al., 2013). For example, PPARγ proteasomal degradation is affected by ligand binding via ligand-dependent changes in ubiquination (Hauser et al., 2000; Li et al., 2016). In this way, agonist-specific PPARγ structures could lead to agonist-specific rates of ubiquination and degradation.

Our findings emphasize that the results of activating nuclear receptors, such as PPARγ, can depend uniquely on the agonist and that several ligands that produce biased coactivator peptide and RID recruitment also produce biased signaling. Further work is needed to determine whether such ligands also cause biased endogenous coactivator recruitment in cells or produce biased signaling via another mechanism.

## Abbreviations

CBP: CREB-binding protein
DMEM: Dulbecco’s modified Eagle’s medium
DR1: direct repeat 1
FL: full length
KEGG: Kyoto Encyclopedia of Genes and Genomes
LBD: ligand binding domain
MED1: mediator of RNA polymerase II transcription subunit 1
MOPS: 4-morpholinepropanesulfonic acid
PGC1α: peroxisome proliferator-activated receptor gamma coactivator-related protein 1
PPARγ: peroxisome proliferator-activated receptor gamma
PPRE: peroxisome proliferator response element
RID: receptor interaction domain
RNA-seq: RNA sequencing
RXRα: retinoic acid receptor alpha
TCEP: tris(2-carboxyethyl)phosphine
TZD: thiazolidinedione

## References

[B1] Acton JJ Black RM Jones AB Moller DE Colwell L Doebber TW Macnaul KL Berger J Wood HB (2005) Benzoyl 2-methyl indoles as selective PPARγ modulators. Bioorg Med Chem Lett 15**:** 357–362. 15603954 10.1016/j.bmcl.2004.10.068

[B2] Ahmadian M Suh JM Hah N Liddle C Atkins AR Downes M Evans RM (2013) PPARgamma signaling and metabolism: the good, the bad and the future. Nat Med 19**:** 557–566. 23652116 10.1038/nm.3159 PMC3870016

[B3] Akdel M Pires DEV Pardo EP Jänes J Zalevsky AO Mészáros B Bryant P Good LL Laskowski RA Pozzati G, (2022) A structural biology community assessment of AlphaFold2 applications. Nat Struct Mol Biol 29**:** 1056–1067. 36344848 10.1038/s41594-022-00849-w PMC9663297

[B4] Auld DS Farmen MW Kahl S (2012) Receptor binding assays for HTS and drug discovery. in Assay Guidance Manual (Markossian S, Grossman A, and Arkin M eds) pp 129–177, Eli Lilly & Company and the National Center for Advancing Translational Sciences, Bethesda (MD). 22553864

[B5] Berger JP Petro AE Macnaul KL Kelly LJ Zhang BB Richards K Elbrecht A Johnson BA Zhou G Doebber TW, (2003) Distinct properties and advantages of a novel peroxisome proliferator-activated protein [gamma] selective modulator. Mol Endocrinol 17**:** 662–676. 12554792 10.1210/me.2002-0217

[B6] Bi R Liu P (2016) Sample size calculation while controlling false discovery rate for differential expression analysis with RNA-sequencing experiments. BMC Bioinformatics 17**:** 146–113. 27029470 10.1186/s12859-016-0994-9 PMC4815167

[B7] Biggin MD (2011) Animal transcription networks as highly connected, quantitative continua. Dev Cell 21**:** 611–626. 22014521 10.1016/j.devcel.2011.09.008

[B8] Broekema MF Massink MPG Donato C de Ligt J Schaarschmidt J Borgman A Schooneman MG Melchers D Gerding MN Houtman R, (2019) Natural helix 9 mutants of PPARγ differently affect its transcriptional activity. Mol Metab 20**:** 115–127. 30595551 10.1016/j.molmet.2018.12.005 PMC6358588

[B9] Bruning JB Chalmers MJ Prasad S Busby SA Kamenecka TM He Y Nettles KW Griffin PR (2007) Partial agonists activate PPARgamma using a helix 12 independent mechanism. Structure 15**:** 1258–1271. 17937915 10.1016/j.str.2007.07.014

[B10] Burris TP de Vera IMS Cote I Flaveny CA Wanninayake US Chatterjee A Walker JK Steinauer N Zhang J Coons LA, (2023) International Union of Basic and Clinical Pharmacology CXIII: Nuclear receptor superfamily—update 2023. Pharmacol Rev 75**:** 1233–1318. 37586884 10.1124/pharmrev.121.000436 PMC10595025

[B11] Burris TP Solt LA Wang Y Crumbley C Banerjee S Griffett K Lundasen T Hughes T Kojetin DJ (2013) Nuclear receptors and their selective pharmacologic modulators. Pharmacol Rev 65**:** 710–778. 23457206 10.1124/pr.112.006833 PMC11060414

[B12] Choi JH Banks AS Estall JL Kajimura S Boström P Laznik D Ruas JL Chalmers MJ Kamenecka TM Blüher M, (2010) Anti-diabetic drugs inhibit obesity-linked phosphorylation of PPARγ by Cdk5. Nature 466**:** 451–456. 20651683 10.1038/nature09291 PMC2987584

[B13] Choi JH Banks AS Kamenecka TM Busby SA Chalmers MJ Kumar N Kuruvilla DS Shin Y He Y Bruning JB, (2011) Antidiabetic actions of a non-agonist PPARγ ligand blocking Cdk5-mediated phosphorylation. Nature 477**:** 477–481. 21892191 10.1038/nature10383 PMC3179551

[B14] Chrisman IM Nemetchek MD de Vera IMS Shang J Heidari Z Long Y Reyes-Caballero H Galindo-Murillo R Cheatham TE Blayo A-L, (2018) Defining a conformational ensemble that directs activation of PPARγ. Nat Commun 9**:** 1794. 29728618 10.1038/s41467-018-04176-x PMC5935666

[B15] Cohen J (1988) Statistical Power Analysis for the Behavioral Sciences, 2nd ed., Routledge, New York.

[B16] Danecek P Bonfield JK Liddle J Marshall J Ohan V Pollard MO Whitwham A Keane T McCarthy SA Davies RM, (2021) Twelve years of SAMtools and BCFtools. Gigascience 10**:** giab008. 33590861 10.1093/gigascience/giab008 PMC7931819

[B17] Davies MJ D’Alessio DA Fradkin J Kernan WN Mathieu C Mingrone G Rossing P Tsapas A Wexler DJ Buse JB (2018) Management of hyperglycemia in type 2 diabetes, 2018. A consensus report by the American Diabetes Association (ADA) and the European Association for the Study of Diabetes (EASD). Diabetes Care 41**:** 2669–2701. 30291106 10.2337/dci18-0033 PMC6245208

[B18] de Vera IMS Zheng J Novick S Shang J Hughes TS Brust R Munoz-Tello P Gardner WJ Marciano DP Kong X, (2017) Synergistic regulation of coregulator/nuclear receptor interaction by ligand and DNA. Structure 25**:** 1506–1518.e4. 28890360 10.1016/j.str.2017.07.019 PMC5653230

[B19] Fagerberg L Hallström BM Oksvold P Kampf C Djureinovic D Odeberg J Habuka M Tahmasebpoor S Danielsson A Edlund K, (2014) Analysis of the human tissue-specific expression by genome-wide integration of transcriptomics and antibody-based proteomics. Mol Cell Proteomics 13**:** 397–406. 24309898 10.1074/mcp.M113.035600 PMC3916642

[B20] Ferrie JJ Karr JP Tjian R Darzacq X (2022) “Structure”-function relationships in eukaryotic transcription factors: the role of intrinsically disordered regions in gene regulation. Mol Cell 82**:** 3970–3984. 36265487 10.1016/j.molcel.2022.09.021

[B21] Haakonsson AK Stahl Madsen M Nielsen R Sandelin A Mandrup S (2013) Acute genome-wide effects of rosiglitazone on PPAR. Mol Endocrinol 27**:** 1536–1549. 23885096 10.1210/me.2013-1080 PMC5415231

[B22] Hauser S Adelmant G Sarraf P Wright HM Mueller E Spiegelman BM (2000) Degradation of the peroxisome proliferator-activated receptor γ is linked to ligand-dependent activation. J Biol Chem 275**:** 18527–18533. 10748014 10.1074/jbc.M001297200

[B23] Heidari Z Chrisman IM Nemetchek MD Novick SJ Blayo A-L Patton T Mendes DE Diaz P Kamenecka TM Griffin PR, (2019) Definition of functionally and structurally distinct repressive states in the nuclear receptor PPARγ. Nat Commun 10**:** 5825. 31862968 10.1038/s41467-019-13768-0 PMC6925260

[B24] Heinz S Benner C Spann N Bertolino E Lin YC Laslo P Cheng JX Murre C Singh H Glass CK (2010) Simple combinations of lineage-determining transcription factors prime cis-regulatory elements required for macrophage and B cell identities. Mol Cell 38**:** 576–589. 20513432 10.1016/j.molcel.2010.05.004 PMC2898526

[B25] Henke BR Blanchard SG Brackeen MF Brown KK Cobb JE Collins JL Harrington WW Hashim MA Hull-Ryde EA Kaldor I, (1998) N -(2-Benzoylphenyl)-l -tyrosine PPARγ agonists. 1. Discovery of a novel series of potent antihyperglycemic and antihyperlipidemic agents. J Med Chem 41**:** 5020–5036. 9836620 10.1021/jm9804127

[B26] Hughes TS Chalmers MJ Novick S Kuruvilla DS Chang MR Kamenecka TM Rance M Johnson BA Burris TP Griffin PR, (2012) Ligand and receptor dynamics contribute to the mechanism of graded PPARγ agonism. Structure 20**:** 139–150. 22244763 10.1016/j.str.2011.10.018 PMC3278220

[B27] Isigkeit L Schallmayer E Busch R Brunello L Menge A Elson L Müller S Knapp S Stolz A Marschner JA, (2024) Chemogenomics for NR1 nuclear hormone receptors. Nat Commun 15**:** 5201. 38890295 10.1038/s41467-024-49493-6 PMC11189487

[B28] Karolchik D Hinrichs AS Furey TS Roskin KM Sugnet CW Haussler D Kent WJ (2004) The UCSC Table Browser data retrieval tool. Nucleic Acids Res 32**:** D493–D496. 14681465 10.1093/nar/gkh103 PMC308837

[B29] Kim D Paggi JM Park C Bennett C Salzberg SL (2019) Graph-based genome alignment and genotyping with HISAT2 and HISAT-genotype. Nat Biotechnol 37**:** 907–915. 31375807 10.1038/s41587-019-0201-4 PMC7605509

[B30] Klein IA Boija A Afeyan LK Hawken SW Fan M Dall'Agnese A Oksuz O Henninger JE Shrinivas K Sabari BR, (2020) Partitioning of cancer therapeutics in nuclear condensates. Science (1979) 368**:** 1386–1392. 10.1126/science.aaz4427 PMC7735713 32554597

[B31] Kolb P Kenakin T Alexander SPH Bermudez M Bohn LM Breinholt CS Bouvier M Hill SJ Kostenis E Martemyanov K, (2022) Community guidelines for GPCR ligand bias: IUPHAR review XX. Br J Pharmacol 179:3651–3674. 35106752 10.1111/bph.15811 PMC7612872

[B32] Lam VQ Zheng J Griffin PR (2017) Unique interactome network signatures for peroxisome proliferator-activated receptor gamma (PPARγ) modulation by functional selective ligands. Mol Cell Proteomics 16**:** 2098–2110. 28972081 10.1074/mcp.RA117.000308 PMC5724174

[B33] Lehmann JM Moore LB Smith-Oliver TA Wilkison WO Willson TM Kliewer SA (1995) An antidiabetic thiazolidinedione is a high affinity ligand for peroxisome proliferator-activated receptor gamma (PPAR gamma). J Biol Chem 270**:** 12953–12956. 7768881 10.1074/jbc.270.22.12953

[B34] Li JJ Wang R Lama R Wang X Floyd ZE Park EA Liao F-F (2016) Ubiquitin ligase NEDD4 Regulates PPAR γ stability and adipocyte differentiation in 3T3-L1 cells. Sci Rep 6**:** 38550. 27917940 10.1038/srep38550 PMC5137149

[B35] Li P Fan W Xu J Lu M Yamamoto H Auwerx J Sears DD Talukdar S Oh D Chen A, (2011) Adipocyte NCoR knockout decreases PPARγ phosphorylation and enhances PPARγ activity and insulin sensitivity. Cell 147**:** 815–826. 22078880 10.1016/j.cell.2011.09.050 PMC3783197

[B36] Liu W Lau F Liu K Wood HB Zhou G Chen Y Li Y Akiyama TE Castriota G Einstein M, (2011) Benzimidazolones: a new class of selective peroxisome proliferator- activated receptor γ (PPARγ) modulators. J Med Chem 54**:** 8541–8554. 22070604 10.1021/jm201061j

[B37] Livernois AM Hnatchuk DJ Findlater EE Graether SP (2009) Obtaining highly purified intrinsically disordered protein by boiling lysis and single step ion exchange. Anal Biochem 392**:** 70–76. 19464251 10.1016/j.ab.2009.05.023

[B38] Loke YK Singh S Furberg CD (2009) Long-term use of thiazolidinediones and fractures in type 2 diabetes: a meta-analysis. CMAJ 180**:** 32–39. 19073651 10.1503/cmaj.080486 PMC2612065

[B39] Love MI Huber W Anders S (2014) Moderated estimation of fold change and dispersion for RNA-seq data with DESeq2. Genome Biol 15**:** 550–521. 25516281 10.1186/s13059-014-0550-8 PMC4302049

[B40] Ma L Gao Z Wu J Zhong B Xie Y Huang W Lin Y (2021) Co-condensation between transcription factor and coactivator p300 modulates transcriptional bursting kinetics. Mol Cell 81**:** 1682–1697.e7. 33651988 10.1016/j.molcel.2021.01.031

[B41] Madsen MS Broekema MF Madsen MR Koppen A Borgman A Gräwe C Thomsen EGK Westland D Kranendonk MEG Koerkamp MG, (2022) PPARγ lipodystrophy mutants reveal intermolecular interactions required for enhancer activation. Nat Commun 13**:** 7090. 36402763 10.1038/s41467-022-34766-9 PMC9675755

[B42] Meylan P Pich C Winkler C Ginster S Mury L Sgandurra M Dreos R Frederick DT Hammond M Boland GM, (2021) Low expression of the PPARγ-regulated gene thioredoxin-interacting protein accompanies human melanoma progression and promotes experimental lung metastases. Sci Rep 11**:** 7847–7817. 33846376 10.1038/s41598-021-86329-5 PMC8042115

[B43] Milo R Jorgensen P Moran U Weber G Springer M (2009) BioNumbers: the database of key numbers in molecular and cell biology. Nucleic Acids Res 38**:** D750–D753. 19854939 10.1093/nar/gkp889 PMC2808940

[B44] Motani A Wang Z Weiszmann J McGee LR Lee G Liu Q Staunton J Fang Z Fuentes H Lindstrom M, (2009) INT131: a selective modulator of PPARγ. J Mol Biol 386**:** 1301–1311. 19452630 10.1016/j.jmb.2009.01.025

[B45] Mouchiroud L Eichner LJ Shaw RJ Auwerx J (2014) Transcriptional coregulators: fine-tuning metabolism. Cell Metab 20**:** 26–40. 24794975 10.1016/j.cmet.2014.03.027 PMC4079747

[B46] Narita T Ito S Higashijima Y Chu WK Neumann K Walter J Satpathy S Liebner T Hamilton WB Maskey E, (2021) Enhancers are activated by p300/CBP activity-dependent PIC assembly, RNAPII recruitment, and pause release. Mol Cell 81**:** 2166–2182.e6. 33765415 10.1016/j.molcel.2021.03.008

[B47] Nassar LR Barber GP Benet-Pagès A Casper J Clawson H Diekhans M Fischer C Gonzalez JN Hinrichs AS Lee BT, (2023) The UCSC Genome Browser database: 2023 update. Nucleic Acids Res 51**:** D1188–D1195. 36420891 10.1093/nar/gkac1072 PMC9825520

[B48] Nemetchek MD Chrisman IM Rayl ML Voss AH Hughes TS (2022) A structural mechanism of nuclear receptor biased agonism. Proc Natl Acad Sci USA 119**:** 2017. 10.1073/pnas.2215333119 PMC9897460 36469765

[B49] Pertea G Pertea M (2020) GFF Utilities: GffRead and GffCompare. F1000Res 9. 10.12688/f1000research.23297.1 PMC7222033 32489650

[B50] Pertea M Kim D Pertea GM Leek JT Salzberg SL (2016) Transcript-level expression analysis of RNA-seq experiments with HISAT, StringTie and Ballgown. Nat Protoc 11**:** 1650–1667. 27560171 10.1038/nprot.2016.095 PMC5032908

[B51] Pertea M Pertea GM Antonescu CM Chang T-C Mendell JT Salzberg SL (2015) StringTie enables improved reconstruction of a transcriptome from RNA-seq reads. Nat Biotechnol 33**:** 290–295. 25690850 10.1038/nbt.3122 PMC4643835

[B52] Rajagopal S Ahn S Rominger DH Gowen-MacDonald W Lam CM Dewire SM Violin JD Lefkowitz RJ (2011) Quantifying ligand bias at seven-transmembrane receptors. Mol Pharmacol 80**:** 367–377. 21610196 10.1124/mol.111.072801 PMC3164332

[B53] Rauluseviciute I Riudavets-Puig R Blanc-Mathieu R Castro-Mondragon JA Ferenc K Kumar V Lemma RB Lucas J Chèneby J Baranasic D, (2024) JASPAR 2024: 20th anniversary of the open-access database of transcription factor binding profiles. Nucleic Acids Res 52**:** D174–D182. 37962376 10.1093/nar/gkad1059 PMC10767809

[B54] Runge-Morris M Kocarek TA Falany CN (2013) Regulation of the cytosolic sulfotransferases by nuclear receptors. Drug Metab Rev 45**:** 15–33. 23330539 10.3109/03602532.2012.748794 PMC3831883

[B55] Santos R Ursu O Gaulton A Bento AP Donadi RS Bologa CG Karlsson A Al-Lazikani B Hersey A Oprea TI, (2017) A comprehensive map of molecular drug targets. Nat Rev Drug Discov 16**:** 19–34. 27910877 10.1038/nrd.2016.230 PMC6314433

[B56] Shang J Kojetin DJ (2021) Structural mechanism underlying ligand binding and activation of PPARγ. Structure 29**:** 940–950. 33713599 10.1016/j.str.2021.02.006 PMC8418994

[B57] Soccio RE Chen ER Lazar MA (2014) Thiazolidinediones and the promise of insulin sensitization in type 2 diabetes. Cell Metab 20**:** 573–591. 25242225 10.1016/j.cmet.2014.08.005 PMC4192012

[B58] Step SE Lim H-W Marinis JM Prokesch A Steger DJ You S-H Won K-J Lazar MA (2014) Anti-diabetic rosiglitazone remodels the adipocyte transcriptome by redistributing transcription to PPARγ-driven enhancers. Genes Dev 28**:** 1018–1028. 24788520 10.1101/gad.237628.114 PMC4018489

[B59] Sundahl N Bridelance J Libert C De Bosscher K Beck IM (2015) Selective glucocorticoid receptor modulation: new directions with non-steroidal scaffolds. Pharmacol Ther 152**:** 28–41. 25958032 10.1016/j.pharmthera.2015.05.001

[B60] Tan Y Muise ES Dai H Raubertas R Wong KK Thompson GM Wood HB Meinke PT Lum PY Thompson JR, (2012) Novel transcriptome profiling analyses demonstrate that selective peroxisome proliferator-activated receptor γ (PPARγ) modulators display attenuated and selective gene regulatory activity in comparison with PPARγ full agonists. Mol Pharmacol 82**:** 68–79. 22496518 10.1124/mol.111.076679

[B61] Tontonoz P Spiegelman BM (2008) Fat and beyond: the diverse biology of PPARgamma. Annu Rev Biochem 77**:** 289–312. 18518822 10.1146/annurev.biochem.77.061307.091829

[B62] Weikum ER Liu X Ortlund EA (2018) The nuclear receptor superfamily: a structural perspective. Protein Science 27**:** 1876–1892. 30109749 10.1002/pro.3496 PMC6201731

[B63] Wickha H (2016) ggplot2: Elegant Graphics for Data Analysis, Springer-Verlag New York.

[B64] Wu T Hu E Xu S Chen M Guo P Dai Z Feng T Zhou L Tang W Zhan L, (2021) clusterProfiler 4.0: a universal enrichment tool for interpreting omics data. The Innovation 2**:** 100141. 34557778 10.1016/j.xinn.2021.100141 PMC8454663

[B65] Yu G Wang L-G Han Y He Q-Y (2012) clusterProfiler: an R Package for Comparing Biological Themes Among Gene Clusters. OMICS 16**:** 284–287. 22455463 10.1089/omi.2011.0118 PMC3339379

[B66] Yu G Wang L-G Yan G-R He Q-Y (2015) DOSE: an R/Bioconductor package for disease ontology semantic and enrichment analysis. Bioinformatics 31**:** 608–609. 25677125 10.1093/bioinformatics/btu684

